# Heat shock protein 90 inhibitor RGRN-305 potently attenuates skin inflammation

**DOI:** 10.3389/fimmu.2023.1128897

**Published:** 2023-02-07

**Authors:** Hakim Ben Abdallah, Sabine Seeler, Anne Bregnhøj, Gautam Ghatnekar, Lasse S. Kristensen, Lars Iversen, Claus Johansen

**Affiliations:** ^1^ Department of Dermatology and Venereology, Aarhus University Hospital, Aarhus, Denmark; ^2^ Department of Biomedicine, Aarhus University, Aarhus, Denmark; ^3^ Regranion, Mount Pleasant, SC, United States

**Keywords:** HSP90 (heat shock protein 90), novel therapeutic strategy, inflammation, small molecule, 12-O-Tetradecanoylphorbol-13-acetate, keratinocytes, mouse model

## Abstract

**Introduction:**

Chronic inflammatory skin diseases may have a profound negative impact on the quality of life. Current treatment options may be inadequate, offering an unsatisfactory response or side effects. Therefore, ongoing efforts exist to identify novel effective and safe treatments. Heat shock protein (HSP) 90 is a chaperone that promotes the activity of a wide range of client proteins including key proinflammatory molecules involved in aberrant inflammation. Recently, a proof-of-concept clinical trial of 13 patients suggested that RGRN-305 (an HSP90 inhibitor) may be an oral treatment for psoriasis. However, HSP90 inhibition may be a novel therapeutic approach extending beyond psoriasis to include multiple immune-mediated inflammatory skin diseases.

**Methods:**

This study aimed to investigate (*i*) the anti-inflammatory effects and mechanisms of HSP90 inhibition and (*ii*) the feasibility of topical RGRN-305 administration (new route of administration) in models of inflammation elicited by 12-O-tetradecanoylphorbol-13-acetate (TPA) in primary human keratinocytes and mice (irritative dermatitis murine model).

**Results/Discussion:**

In primary human keratinocytes stimulated with TPA, a Nanostring® nCounter gene expression assay demonstrated that HSP90 inhibition with RGRN-305 suppressed many proinflammatory genes. Furthermore, when measured by quantitative real-time polymerase chain reaction (RT-qPCR), RGRN-305 significantly reduced the gene expression of *TNF, IL1B, IL6* and *CXCL8*. We next demonstrated that topical RGRN-305 application significantly ameliorated TPA-induced skin inflammation in mice. The increase in ear thickness (a marker of inflammation) was significantly reduced (up to 89% inhibition). In accordance, RT-qPCR of the ear tissue demonstrated that RGRN-305 robustly reduced the gene expression of proinflammatory markers (*Tnf, Il1b, Il6, Il17A* and *Defb4*). Moreover, RNA sequencing revealed that RGRN-305 mitigated TPA-induced alterations in gene expression and suppressed genes implicated in inflammation. Lastly, we discovered that the anti-inflammatory effects were mediated, at least partly, by suppressing the activity of NF-κB, ERK1/2, p38 MAPK and c-Jun signaling pathways, which are consistent with previous findings in other experimental models beyond skin inflammation. In summary, HSP90 inhibition robustly suppressed TPA-induced inflammation by targeting key proinflammatory cytokines and signaling pathways. Our findings suggest that HSP90 inhibition may be a novel mechanism of action for treating immune-mediated skin disease beyond psoriasis, and it may be a topical treatment option.

## Introduction

Inflammation is a biological response of the immune system that implicates a complex interplay between different cell types, chemokines, cytokines and intracellular signaling pathways (1, 2). Dysregulated inflammation underlies the pathogenesis of multiple inflammatory skin diseases (e.g., psoriasis, atopic dermatitis and hidradenitis suppurativa) that cause significantly reduced quality of life for the patients and constitute a substantial burden on the healthcare system (3). Recent progress in research on highly effective biologics that target cytokines has led to breakthroughs in the management of inflammatory skin diseases such as psoriasis (4). Nonetheless, some groups of patients do not attain or maintain a satisfactory response (5). Disadvantages of biologics compared with small molecules include parenteral administration, high manufacturing cost and risk of immunogenicity (6). Thus, a pressing need exists for novel small molecules that may fill the remaining gaps in the repertoire of therapeutics for inflammatory skin diseases.

Heat shock proteins (HSP) are a group of chaperone molecules whose major function is the maintenance of cellular homeostasis by folding and promoting the function of endogenous proteins (referred to as ‘‘client proteins’’) (7). The heat shock protein 90 (HSP90) is the most abundant HSP (4-6% of total protein during cellular stress); it exists in two cytosolic isoforms (HSP90α and HSP90β) and two organelle-specific isoforms including glucose-regulated protein 94 (GRP94/gp96) localized in the endoplasmic reticulum and tumor necrosis factor receptor-associated protein 1 (TRAP1) localized in the mitochondria (8). HSP90 consists of three conserved domains: an N-terminal domain where the ATP-binding site is located (an ATPase activity is required for its chaperone function); a middle domain which is involved in binding to client proteins: and a C-terminal domain, which is responsible for HSP90 dimerization (9). Several co-chaperones (e.g., HSP70, CDC37 and AHA1) modulate the ATPase activity or functional range of client proteins (10). HSP90 is sometimes referred to as the ‘signal transduction chaperone’ given a large number of kinases and transcription factors being client proteins (11–13). Numerous of these client proteins are involved in key inflammatory pathways (14, 15). Thus, HSP90 inhibition may target these client proteins involved in inflammation, providing a rationale as a treatment for multiple inflammatory skin conditions. HSP90 inhibition has been shown to mediate anti-inflammatory effects in inflammatory models such as rheumatoid arthritis and systemic lupus erythematous (16–21). Moreover, HSP90 has been implicated in wound healing and skin cell motility (22, 23).

RGRN-305 (formerly known as Debio 0932 and CUDC-305) is an imidazopyridine derivative HSP90 inhibitor displaying a high affinity to the N-terminal ATP-binding site for HSP90α and HSP90β, and thereby blocks the ATPase activity and chaperone function (24). RGRN-305 has been shown to exhibit anti-inflammatory effects in preclinical psoriasis studies (21, 25). Recently, the potential of oral RGRN-305 treatment in psoriasis was supported by a proof-of-concept clinical trial (26). Given the several anti-inflammatory mechanisms revealed by the transcriptome analysis (26), the benefits of HSP90 inhibition may extend beyond psoriasis to include other inflammatory skin diseases.

HSP90 inhibition has, until now, not been evaluated in chemical-mediated inflammation induced by 12-O-tetradecanoylphorbol-13-acetate (TPA). It is a widely used phorbol ester activating protein kinase C, a central signaling molecule downstream many inflammatory receptors, driving inflammatory cascades involving proinflammatory mediators such as TNF, IL-1β and IL-6, which has been linked to many inflammatory skin diseases (27, 28).

Therefore, this study aimed to investigate (*i*) the anti-inflammatory effects and mechanisms of HSP90 inhibition, and (*ii*) the feasibility of topical RGRN-305 administration (new route of administration) using models of inflammation induced by TPA in primary human keratinocytes and a murine model for skin inflammation.

## Materials and methods

### HSP90 inhibitor RGRN-305

The HSP90 inhibitor RGRN-305 (molecular weight = 442.58 g/mol) was kindly provided by Regranion (Mount Pleasant, SC, USA).

### Cell culture

Primary normal human epidermal keratinocytes were obtained from skin samples of six healthy adults undergoing plastic surgery as previously described (29). The keratinocytes were seeded in 6-well plates and cultured in Keratinocyte SFM (Gibco, Thermo Fisher Scientific, Waltham, MA, USA) supplemented with growth factors (Gibco) and 5 µg/mL Gentamicin (Gibco) at 37°C and 5% CO_2_ in a humidified incubator until 60-70% confluency. The medium was changed to basal medium (same as before without growth factors) for 24 hours before experiments were initiated. The keratinocytes were preincubated with RGRN-305 (5 µM) or drug-vehicle (water) for 8 hours before stimulation with 100 nM TPA (Sigma-Aldrich, Burlington, MA, USA) or vehicle (DMSO) for up to 24 hours.

### Isolation of RNA from primary human keratinocytes

The cells were washed twice with ice-cold phosphate-buffered saline (PBS) (Gibco) followed by the addition of SV RNA lysis buffer (Promega, Madison, WI, USA). Total RNA was isolated using the SV 96 Total RNA Isolation System (Promega) according to the manufacturer’s instructions. The RNA concentration and purity were assessed by NanoDrop™ 2000 Spectrophotometer (ThermoFisher). Samples were stored at -80°C until further use.

### Reverse transcription-quantitative PCR

Total RNA was diluted to 10 ng/µL across all samples before RNA was reversely transcribed to cDNA using TaqMan™ Reverse Transcription Reagents (ThermoFisher) and Peltier Thermal Cycler-200 (MJ Research Inc. Waltham, MA, USA) according to the manufacturer’s instructions. Real-time PCR was performed with StepOnePlus Real-Time PCR system (Applied Biosystems™, Waltham, MA, USA) using TaqMan™ Universal PCR Master Mix (ThermoFisher) and TaqMan™ primers and probes for *TNF* (Hs00174128_m1), *Tnf* (Mm00443258_m1), *IL1B* (Hs01555410_m1), *Il1b* (Mm00434228_m1), *Il6* (Mm00446190_m1), *CXCL8* (Hs00174103_m1), *Il17a* (Mm00439618_m1), Defb4 (Mm00731768_m1), RPLP0 (Hs99999902_m1) and *Gapdh* (Mm99999915_m1) according to the manufacturer’s instructions. The real-time PCR consisted of 2 initial steps (2 min at 50°C and 10 min at 95°C) followed by 40 PCR cycles (15 seconds at 95°C and 1 min at 60°C). The genes were analyzed in triplicates and the relative gene expression was quantified by the relative standard curve method using StepOne™ Software v2.1 (30). *RPLP0* or *Gapdh* was used as reference genes to obtain normalized expression values that were shown as fold changes relative to the levels in unstimulated and untreated samples (vehicle).

### NanoString nCounter gene expression

For each sample, 100 ng of total RNA from keratinocytes was hybridized for 19.5 hours with probes from the nCounter Human Immunology V2 Panel containing 579 target genes and 15 reference genes (NanoString Technologies, Seattle, WA, USA). The samples were examined using nCounter SPRINT profiler (NanoString Technologies) according to the manufacturer’s instructions. Before the differential expression analyses, the raw data were normalized using nSOLVER 4.0 (NanoString Technologies) by performing background subtraction, positive control normalization and normalization to housekeeping genes (*EEF1G, GAPDH, GUSB, OAZ1*, and *RPL19)*. Genes were filtered to only include genes with an average normalized count ≥ 20 corresponding to 271 genes. Differential gene expression analysis was performed by multiple paired t-tests with a false discovery rate < 0.05 using the two-stage step-up method of Benjamini, Krieger and Yekutiel in GraphPad Prism 9.0 software (31).

### Isolation of protein from human keratinocytes

Keratinocytes were added cell lysis buffer containing phenylmethylsulfonyl fluoride and Complete Protease Inhibitor Cocktail (Sigma-Aldrich) followed by centrifugation at 13.000 g for 3 minutes and collection of the supernatant.

### Western blotting

An equal amount of protein (20 µg as determined by Bradford Protein Assay) for each sample was separated by gel electrophoresis using 10% Mini-PROTEAN TGX Precast Protein Gels (Bio-Rad, Hercules, CA, USA) and Mini Trans-Blot Cell (Bio-Rad). The proteins were blotted onto nitrocellulose membranes (Bio-Rad; catalog#1620146) by Trans-Blot Turbo Transfer System (Bio-Rad). The membranes were then incubated overnight at 4°C with the primary antibodies as follows (catalog#): anti-p-p65 (3033), anti-p-c-Jun (9261), anti-p-p38 (9211) and p-ERK1/2 (9101) purchased from Cell Signaling Technology (Danvers, MA, USA). The primary antibodies were detected by 1-hour incubation at room temperature using the secondary antibody anti-rabbit immunoglobulin G (IgG)-HRP (catalog#7074; Cell Signaling Technology). Protein bands were visualized using Clarity Western ECL Substrate (Bio-Rad) and C-DiGit Blot Scanner (LI-COR, Lincoln, NE, USA). Following which, the membranes were stripped and reprobed with β-actin (catalog# A-1978; Sigma-Aldrich) and anti-mouse IgG-HRP (catalog# p0447; Dako, Glostrup, Denmark). Densitometric analysis of the band and background intensities were measured by Image Studio Digits Version 3.1. The results were normalized to the β-actin levels and shown as fold changes relative to the unstimulated and untreated samples (vehicle).

### Experimental animals

Female C57BL/6J mice (6-8 weeks old) were purchased from Taconic (Ejby, Denmark). The mice were housed in animal facilities with a temperature of 19-25°C and a 12-hour light/dark cycle. The mice had access to laboratory rodent food and water *ad libitum* and were acclimated for 1 week before the experiments.

### TPA-induced ear inflammation

Mice were randomly assigned into 4 different groups to receive: (1) Vehicle; (2) RGRN-305; (3) TPA; or (4) RGRN-305 + TPA. Two independent experiments were conducted. RGRN-305 (50 mg/mL) and TPA (125 µg/mL) were freshly dissolved in acetone for each experiment. Topical application (20 µL) on the ear with RGRN-305 (~1 mg) or acetone was applied 100 minutes before challenge with 20 µL of TPA (~2.5 µg) or acetone. Ear thickness was measured at TPA challenge (0 hour) and post-TPA challenge (4 and 8 hours) using a Mitutoyo digimatic caliper. Mice were anesthetized with 2% isoflurane during the experimental procedures. At the end of the study, mice were euthanized by cervical dislocation and biopsies from the ear were collected for further analyses.

### Isolation of RNA from ear biopsies

Four-millimeter ear punch biopsies obtained from mice (4 hours post-TPA challenge) were immediately frozen and stored in liquid nitrogen. The biopsies were transferred to 750 µL of RNAlater-ICE (ThermoFisher) at −80°C cold for 20 minutes and stored overnight at -20°C. Subsequently, the biopsies were transferred to 175 µL of SV RNA lysis buffer added β-mercaptoethanol (SV Total RNA Isolation System; Promega) and homogenized using TissueLyser (Qiagen, Hilden, Germany). The remaining steps of RNA purification, including DNase treatment, were performed according to the manufacturer’s instructions.

### Isolation of protein from ear biopsies

The remaining ears were clipped off and placed in liquid nitrogen. The ears were homogenized in cell lysis buffer containing phenylmethylsulfonyl fluoride and Complete Protease Inhibitor Cocktail (Sigma-Aldrich) using TissueLyser for 2 x 2 minutes. Subsequently, the samples were sonicated 5 x 10 s and centrifuged at 10.000 g for 10 minutes. The supernatant constituted the whole cell protein extract.

### Histology

Four-millimeter punch biopsies from the ears of mice (4 hours post TPA challenge) were immediately fixed in 4% formaldehyde and embedded in paraffin. The paraffin-embedded biopsies were sliced into 4 μm thick sections and stained with hematoxylin and eosin and Ki-67 following standard protocols.

### RNA sequencing

Paired-end RNA sequencing of RNA isolated from ear tissue from mice was performed by Eurofins Genomics Europe Sequencing GmbH (Konstanz, Germany) according to their protocols. RNA sequencing library was prepared with 100 ng of total RNA using NEBNext Ultra II Directional RNA Library Prep Kit for Illumina. mRNA quality measurement was performed using Fragment Analyzer. Sequencing was performed on the Illumina NovaSeq 6000 platform using 2x150 Sequence mode to obtain at least 20 million read pairs. Raw reads were aligned to the mouse reference genome (GRCm39 primary assembly) and gene counts were obtained by uniquely aligned unambiguous reads using the R package Subread (version 2.10.3) (32). Library size correction and variance stabilization were achieved by regularized logarithm transformation (rlog) using the R package DESeq2 (1.36.0) (33). The regularized log-transformed normalized counts were used for downstream analysis such as principal component analysis (PCA) and heat map visualization (pheatmap 1.0.12). Differential gene expression analyses were performed using DESeq2 with independent filtering enabled (33). The Wald test was used for pairwise comparisons. To adjust for multiple testing, the p-values (i.e., q-values) were adjusted by controlling the false discovery rate (FDR) set at 0.05 using the procedure of Benjamini and Hochberg. Gene Ontology (GO) and Kyoto Encyclopedia of Genes and Genomes (KEGG) enrichment analyses were performed with ShinyGO 0.76 (34).

### Statistical analyses

Two-tailed P-values <0.05 were considered statistically significant. Pair-wise comparison of ear thickness, mRNA (qPCR) and protein levels (western blot) in mice and primary keratinocytes were respectively performed by unpaired and paired t-tests if data were normally distributed, otherwise Mann-Whitney and Wilcoxon signed-rank tests were used. The statistical analyses were performed with GraphPad Prism 9.0 and R 4.2.0 software.

### Ethical statement

The Central Jutland Regional Committee on Health Research Ethics approved the experiments with primary human keratinocytes (M-20110027) and consent was obtained from the donors to donate their excessive skin for research purposes.

The Danish Animal Experiments Inspectorate approved the experimental animal procedures (2021–15–0201–00893). The study was carried out in accordance with the Danish Animal Welfare Act for the Care and Use of Animals for Scientific Purposes, and all efforts were made to minimize suffering.

## Results

### RGRN-305 significantly dampened the TPA-induced inflammatory response in primary human keratinocytes

**Figure 1:**
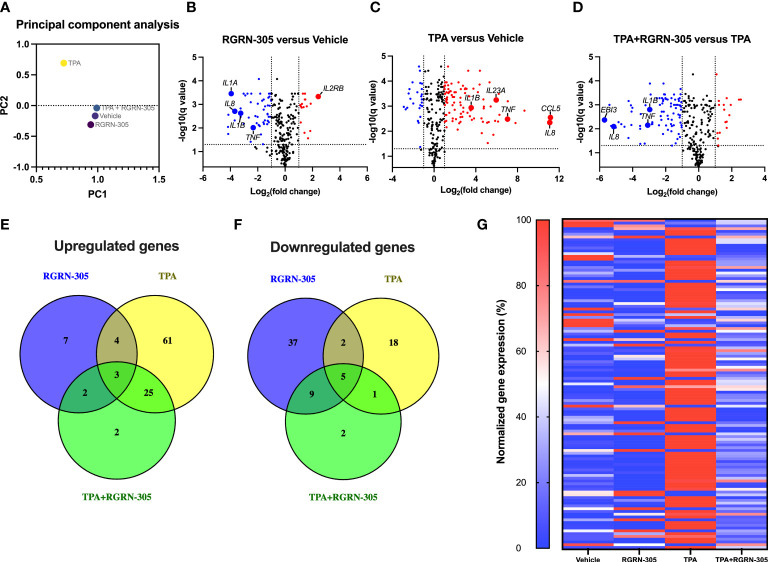
Nanostring nCounter gene expression analysis. Primary human keratinocytes were preincubated with RGRN-305 (5 µM) for 8 hours before stimulation with TPA (100 nM) for 8 hours (n = 6). **(A)** Principal-component analysis (PCA) of the cultured human keratinocytes. **(B–D)** Volcano plots showing log_2_ fold change (x-axis) and q-values (y-axis) comparing the different treatment groups: RGRN-305 versus vehicle **(B)** TPA versus vehicle **(C)** and TPA+RGRN-305 versus TPA **(D)**. The dashed horizontal lines indicate a q-value of 0.05, while the vertical lines mark a log_2_ fold change of 1. Red dots represent upregulated differentially expressed genes (DEGs), whereas blue dots represent downregulated DEGs. **(E, F)** Venn diagrams depicting overlapping upregulated **(E)** and downregulated **(F)** DEGs. **(G)** Heatmap of DEGs upon TPA stimulation. Gene expression was normalized to a scale ranging between 0 and 100%. The smallest and largest values of normalized gene counts across all samples for a given gene were defined as 0 and 100%, respectively. DEGs were defined as at least 1 log_2_ fold change and q-value < 0.05. DEG, differentially expressed gene. TPA, 12-O-Tetradecanoylphorbol-13-acetate.

To investigate the anti-inflammatory effects of RGRN-305 in cultured normal human keratinocytes stimulated with TPA, the mRNA levels of 579 inflammatory genes were analyzed with Nanostring nCounter Human Immunology Panel. The PCA plot (**Figure 1A**) revealed a distinct and separated transcriptional signature for keratinocytes stimulated with TPA, whereas keratinocytes treated with vehicle, RGRN-305 or RGRN-305 + TPA clustered together. This indicates that RGRN-305 abrogated the inflammatory gene expression induced by TPA. In agreement with the PCA, the volcano plots (**Figures 1B–D**) showed a trend of upregulation of inflammatory genes upon TPA stimulation (TPA versus vehicle) whereas the addition of RGRN-305 treatment led to a trend of downregulation (TPA+RGRN-305 versus TPA). Accordingly, 93 differential expressed genes (DEGs), of which 61 genes including *IL1B*, *CXCL8* and *IL17A* were uniquely upregulated by TPA in comparison to the vehicle group. In contrast, only 32 DEGs were upregulated when adding RGRN-305 treatment including two uniquely upregulated genes (*RUNX1* and *ITGB2*) (**Figure 1E**). RGRN-305 treatment alone without TPA in comparison to the vehicle group led to 7 uniquely upregulated genes and 37 uniquely downregulated DEGs including *TNF*, *IL1B* and *CXCL8* (**Figures 1E, F**). Heatmap of DEGs upon TPA stimulation (119 genes) exhibited a distinct pattern characterized by increased gene expression in keratinocytes stimulated with TPA, whereas keratinocytes treated with RGRN-305 (either alone or with TPA) exhibited a pattern that was more similar to the vehicle group (**Figure 1G**). Lists of all genes with their relative gene expression (fold ratio) and q-values are available in **Table S1**. Taken together, we demonstrated that RGRN-305 robustly inhibited the TPA-induced expression of proinflammatory genes in primary keratinocytes.

**Figure 2:**
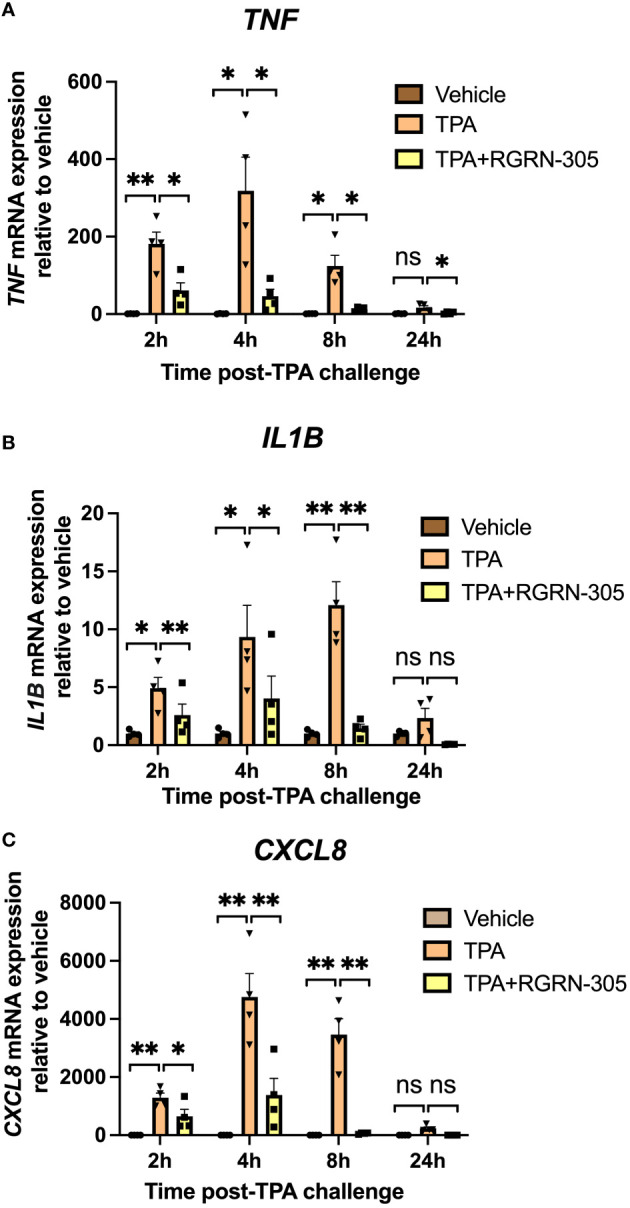
RT-qPCR gene expression analysis of primary human keratinocytes. Effects of RGRN-305 (5 µM) and TPA (100 nM) on mRNA levels were measured at indicated times by RT-qPCR (two independent experiments, n = 4). The mRNA levels of *TNF* **(A)**, *IL1B* **(B)** and *CXCL8* **(C)**, normalized to *RPLP0*, are shown as fold change relative to the vehicle group. Data are shown as mean ± SEM. *p < 0.05, **p ≤0.01. ns, not significant. TPA, 12-O-Tetradecanoylphorbol-13-acetate.

The inhibitory effect of RGRN-305 on proinflammatory cytokines was confirmed by RT-qPCR validating that RGRN-305 significantly suppressed the mRNA levels of *TNF*, *IL1B* and *CXCL8* after 2, 4 and 8 hours of TPA-challenge (**Figure 2**). The tested concentrations of TPA and RGRN-305 were well-tolerated by the keratinocytes (3-6% cytotoxicity; **Figure S1**).

**Figure 3:**
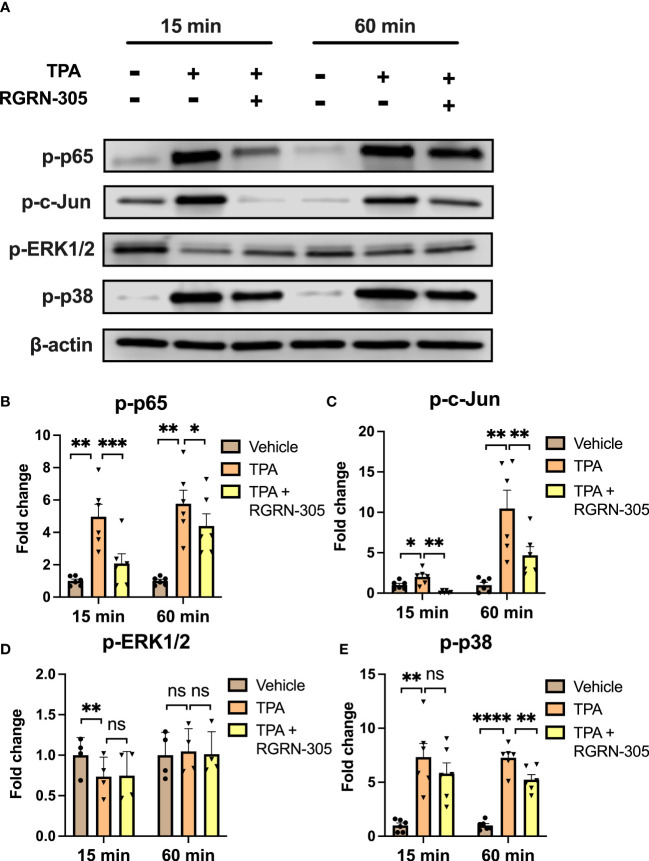
Effects of RGRN-305 on key signaling pathways in primary human keratinocytes. Phosphorylation status of key signaling molecules was determined in primary human keratinocytes preincubated with RGRN-305 (5 µM) and stimulated with TPA (100 nM) for 15 minutes and 60 minutes. **(A)** Representative Western blots and densitometric analyses of p-p65 **(B)**, p-c-Jun **(C)**, p-ERK1/2 **(D)** and p-p38 **(E)**. To normalize protein expression, β-actin was used as a loading control. Data are shown as mean ± SEM fold change relative to vehicle (3 independent experiments, n = 6). *p < 0.05, **p ≤0.01, ***p ≤0.001, ****p ≤ 0.0001. ns, not significant. TPA, 12-O-Tetradecanoylphorbol-13-acetate.

To better understand the molecular mechanisms of RGRN-305, we determined the levels of key phosphorylated/activated signaling proteins (**Figure 3**). TPA increased the phosphorylation status of p65/NF-κB, c-Jun and p38 MAPK, which was significantly decreased by RGRN-305 treatment. The phosphorylation of ERK1/2 was slightly decreased in keratinocytes stimulated with TPA for 15 minutes, whereas no significant effects were observed after 60 minutes (**Figure 3**).

### Topically administered RGRN-305 significantly suppressed TPA-induced ear inflammation in mice

**Figure 4:**
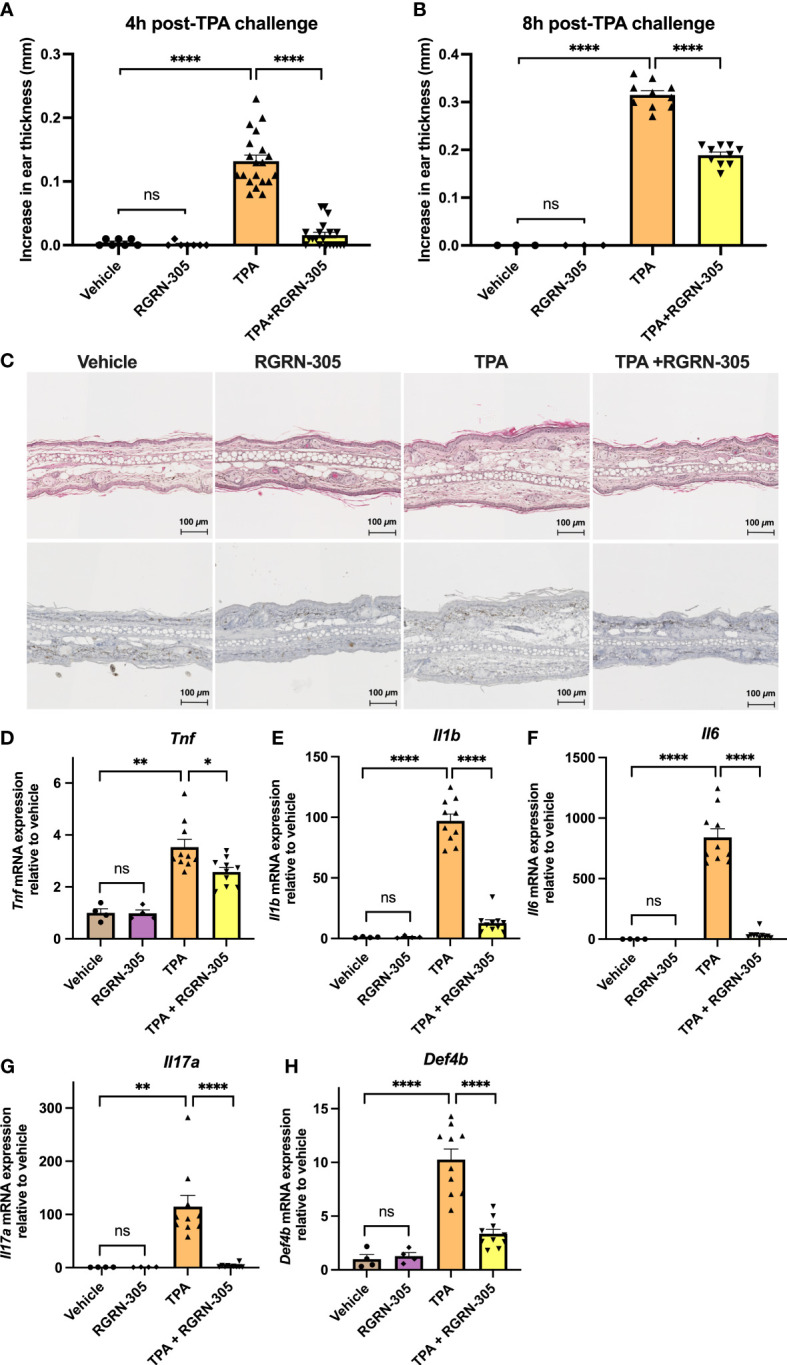
Effects of RGRN-305 on skin inflammation elicited by TPA in mice. C57BL/6 mice were pretreated topically with 1 mg/ear RGRN-305 or vehicle (acetone) 100 minutes before challenge with 2.5 µg TPA or vehicle. Two independent experiments were conducted. Total number of mice in treatment groups: vehicle = 7, RGRN-305 = 7, TPA = 20, TPA+RGRN-305 = 20. **(A, B)** Ear thickness change measured 4 **(A)** and 8 hours **(B)** post-TPA challenge. **(C)** Representative images of hematoxylin and eosin (upper panel) and Ki-67 (lower panel) stained histological sections of mice ears collected 4 hours post-TPA challenge (four to ten mice per treatment group). **(D–H)** mRNA expression of indicated proinflammatory cytokines measured by RT-qPCR in mice ear punch biopsies collected 4 hours post-TPA challenge (four to ten mice per treatment group, n = 28). Data are shown as mean ± SEM fold change relative to vehicle.*p < 0.05, **p ≤0.01, ***p ≤0.001, ****p ≤ 0.0001. TPA, 12-O-Tetradecanoylphorbol-13-acetate.

We next investigated the effect of topically applied RGRN-305 on TPA-induced ear inflammation in C57BL/6 mice. TPA-induced ear swelling was significantly suppressed by RGRN-305 corresponding to 89% and 40% inhibition of ear thickness increase 4 and 8 hours post-TPA application, respectively (**Figures 4A, B**). Similarly, histological analysis of ear tissue displayed substantial dermal oedema in mice treated with TPA, which was mitigated by RGRN-305 treatment (**Figure 4C**). Furthermore, RGRN-305 robustly reduced the TPA-induced mRNA levels of proinflammatory markers (*Tnf*, *Il1b*, *Il6*, *Il17A* and *Defb4*) measured by RT-qPCR (**Figures 4D–H**).

**Figure 5:**
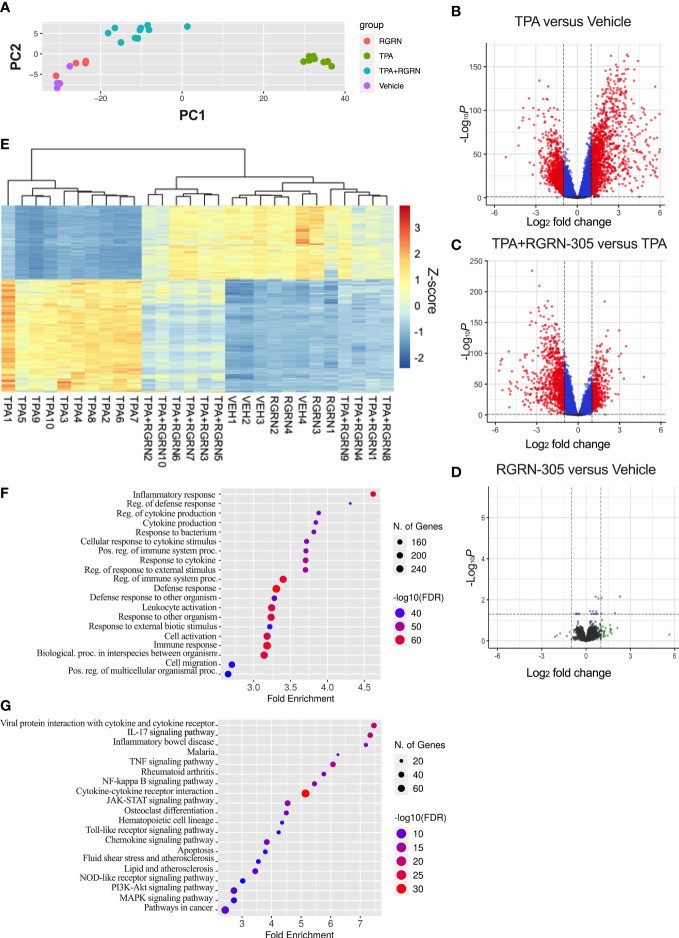
RNA sequencing of ear biopsies from mice 4 hours post-TPA challenge. Number of mice in treatment groups: vehicle = 4, RGRN-305 = 4, TPA = 10, TPA+RGRN-305 = 10). **(A)** Principal component analysis (PCA) performed on regularized log (rlog) transformed normalized counts for the 28 samples. **(B–D)** Volcano plots depicting log_2_ fold change (x-axis) and adjusted p-values (y-axis) comparing the different treatment groups. The dashed horizontal lines indicate a q-value of 0.05, whereas the vertical lines mark a log_2_ fold change of 1. Red dots represent differentially expressed genes (DEGs). Genes were filtered to include those with a base mean > 50 (mean of normalized counts of all samples). **(E)** Heatmap of DEGs upon TPA-induced inflammation (log_2_fold change > 1 and base mean > 50). The gene expression levels are normalized z-score values determined from rlog normalized counts. Rows represent genes, whereas the columns represent each sample. **(F, G)** Enrichment analyses of Gene Ontology biological processes **(F)** and KEGG pathways **(G)** significantly enriched in downregulated DEGs (log_2_fold change < -1) comparing TPA+RGRN-305 versus TPA. The top 20 terms ranked by smallest false discovery rate are presented. DEG, differentially expressed genes. TPA, 12-O-Tetradecanoylphorbol-13-acetate.

To gain further insights into alterations in the transcriptome, RNA sequencing was performed on the ear tissue (**Figure 5**). Samples derived from mice 4 hours post-TPA challenge were chosen given the relatively short half-life of RGRN-305 in different tissues (2-7 hours) (24) and the pronounced effect observed (89% inhibition). The PCA plot revealed that mice treated with vehicle or RGRN-305 alone did cluster and were only slightly separated from the mice treated with TPA+RGRN-305 unlike the distinctly separated pattern of mice challenged with TPA (**Figure 5A**). Likewise, as visually illustrated in the volcano plots, mice challenged with TPA caused a shift of DEGs towards the right indicating upregulation of DEGs compared with vehicle (**Figure 5B**). In contrast, the addition of RGRN-305 treatment to TPA caused a shift of DEGs towards the left, indicating downregulation of genes compared with mice challenged with TPA without RGRN-305 (**Figure 5C**). RGRN-305 alone did not lead to any meaningful changes in gene expression since only 4 genes (*Rpl27-ps1*, *Tnfrsf9*, *Fosl1*, *Epgn*) were differentially expressed (**Figure 5D**). Additionally, in a heatmap with hierarchical clustering (**Figure 5E**), mice treated with RGRN-305+TPA were clustered with mice treated with vehicle or RGRN-305 alone, whereas mice treated solely with TPA were separated. Collectively, our data demonstrate that RGRN-305 mitigated the TPA-induced alterations in gene expression. Lists of all genes in the differential gene expression analyses are available in **Table S2**. Moreover, the RNA sequencing data demonstrated that TPA-induced skin inflammation evoked significant upregulation of *Hsp90aa1* (2.68 fold) and *Hsp90ab1* (1.19 fold), whereas no changes were observed for *Hsp90b1* and *Trap1* gene expression (**Figure S2**).

**Figure 6:**
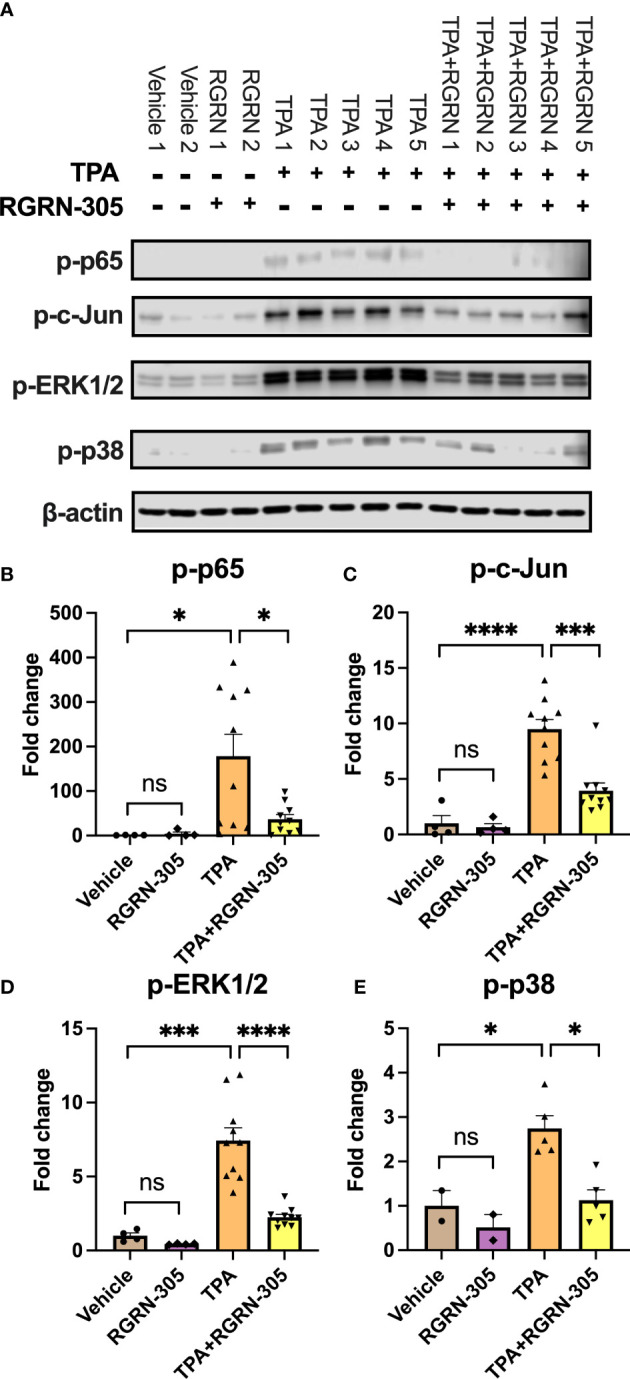
Effects of RGRN-305 on the phosphorylation status of key signaling molecules in mice challenged with TPA post 4 hours. **(A)** Representative Western blots and densitometric analyses of p-p65 **(B)**, p-c-Jun **(C)**, p-ERK1/2 **(D)** and p-p38 **(E)**. β-actin was used as a loading control. Four to ten mice per treatment group. Data are shown as mean ± SEM fold change relative to vehicle. *p < 0.05, **p ≤0.01, ***p ≤0.001, ****p ≤ 0.0001. ns, not significant. TPA, 12-O-Tetradecanoylphorbol-13-acetate.

To further investigate the biological roles of the alterations in gene expression, we conducted a Gene Ontology enrichment analysis of downregulated DEGs comparing mice treated with RGRN-305+TPA versus TPA (**Figure 5F**). Our analyses showed that the majority of the top 20 terms were related to inflammation, indicating that RGRN-305 suppressed genes implicated in inflammation. Furthermore, using KEGG pathways, we detected an overrepresentation of downregulated DEGs in several inflammatory signaling pathways such as NF-κB and MAPK (**Figure 5G**). To further elucidate the molecular effect of RGRN-305, we used Western blot to demonstrate that RGRN-305 treatment significantly decreased TPA-induced phosphorylation/activation of p65/NF-κB, c-Jun, ERK1/2 and p38 MAPK (**Figure 6**).

Taken together, HSP90 inhibition robustly suppressed TPA-induced inflammation by targeting key inflammatory pathways.

## Discussion

In this study, we demonstrated several anti-inflammatory mechanisms of HSP90 inhibition leading to suppression of inflammation induced by TPA in primary human keratinocytes and in a murine model, supporting the hypothesis that HSP90 inhibition alleviates skin inflammation. Additionally, we demonstrated that RGRN-305 may be administered topically, offering advantages given the lower systemic exposure and higher tolerability, which prompts further clinical evaluation in inflammatory skin diseases.

HSP90 inhibitors have been in development (phase I-III) for oncologic indications for several decades, though the research concerning inflammation is relatively scarce (35). Our results are consistent with other animal studies in which HSP90 inhibition suppressed inflammatory pathways and improved the clinical course in inflammatory models (rheumatoid arthritis, systemic lupus erythematous, epidermolysis bullosa acquisita and psoriasis) (16–21). In line with other studies, our results showed that HSP90 inhibition led to inhibition of key proinflammatory cytokines (e.g., TNF, IL-1β, IL-6, IL-8) (16, 17, 25). Furthermore, key signaling pathways implicated in inflammation including NF-kB and MAPK kinase signaling (ERK1/2, p38, JNKs) have been shown to be suppressed by HSP90 inhibition in experimental models within rheumatology, which is consistent with our findings (16, 17, 36, 37). Remarkably, the phosphorylation level of ERK1/2 in our primary human keratinocytes was not increased by TPA or inhibited by RGRN-305. This is likely due to elevated baseline levels mediated by a separate and HSP90-independent pathway as suggested by the abundant expression in the vehicle-treated samples. Nonetheless, the samples from mice showed that p-ERK1/2 was significantly increased by TPA and reduced by RGRN-305 treatment. In accordance, RGRN-305 has been shown to block p-ERK1/2 signaling in cancer cell lines (24).

Given the relatively short half-life of RGRN-305 (2-7 hours), it is not surprising that the efficacy measured as inhibition of the ear thickness increase faded from 89% (4 hours post-TPA challenge) to 40% (8 hours post-TPA challenge; **Figures 4A, B**). Potential strategies to improve the efficacy of RGRN-305 may be frequent drug applications or increasing the half-life and skin permeation by optimized drug formulations.

**Figure 5E** shows two separate clusters of TPA+RGRN-305-treated mice; however, no differences were found in ear thickness reduction or inflammatory markers measured by RT-qPCR. The separation may be caused by random variation due to factors such as scratching or grooming of the ears.

Considering the various functions of HSP90 client proteins, the relevant anti-inflammatory mechanisms of HSP90 as it relates to individual inflammatory skin diseases still need to be fully elucidated. However, our enrichment analyses demonstrated that the KEGG pathway ‘cytokine-cytokine receptor interaction’ was significantly enriched, suggesting that HSP90 inhibition may target cytokine receptors. The pathway diagram illustrated that genes related to cytokine receptors were targeted (**Figure S3**), supporting a mechanism of action encompassing cytokine receptors in addition to proinflammatory cytokines and intracellular signaling molecules.

A recent clinical trial of 13 patients demonstrated that orally dosed RGRN-305 led to improvement of plaque psoriasis (26). In line with our data, the transcriptome analyses indicated that the immunoregulatory effects of HSP90 inhibition were mediated by targeting proinflammatory pathways (25, 26). Other significant pathways in our enrichment analyses included the IL-17 signaling pathway. Considering the prominent role of IL-17 signaling in psoriasis, our findings support the reported beneficial effect of HSP90 inhibition in this disease (26, 38). Moreover, the pathway diagram of IL-17 signaling showed that HSP90 inhibition broadly targeted other clinically relevant cytokines used as targets of anti-inflammatory therapeutics including TNF, IL-1β, IL-4, IL-6, IL-13 and IL-17A (**Figure S4**). Given the broad anti-inflammatory mechanisms, the benefits of HSP90 inhibition may extend beyond psoriasis to include other immune-mediated inflammatory skin diseases. Currently, another clinical trial is investigating the feasibility of orally dosed RGRN-305 in the treatment of hidradenitis suppurativa (NCT05286567).

The suppression of skin inflammation secondary to HSP90 inhibition may be conferred by the inhibition of specific or combinations of HSP90 isoforms. Our RNA sequencing data from mice (**Figure S2**) revealed that TPA-induced skin inflammation significantly upregulated *Hsp90aa1* (2.68 fold) and *Hsp90ab1* (1.19 fold), suggesting that RGRN-305 might mediate its anti-inflammatory effects primarily by inhibition of Hsp90α and to a lesser extent Hsp90β in this inflammation model. However, further research is required to determine the roles of the different HSP90 isoforms in skin inflammation.

While HSP90 functions have been well-described within the cell, extracellular HSP90 (eHSP90) also appears to exert important functions outside the cell (39). Different molecular mechanisms appear to underlie the effects of eHSP90 extending beyond activation of extracellular clients by chaperone activity to include molecular entities of HSP90 conferring effects independent of the chaperone activity (40). In the skin, eHSP90 has been shown to promote wound healing through a non-chaperone function (22, 23), whereas patients with bullous pemphigoid (an autoimmune blistering skin disease) demonstrated increased intracellular and decreased extracellular HSP90 expression compared to healthy volunteers (41). Given the sparsely available information, the role of eHSP90 in skin inflammation remains poorly understood. Thus, further research is warranted as insights into eHSP90 and skin inflammation may support the development of novel drugs targeting HSP90.

In summary, HSP90 inhibition by RGRN-305 significantly ameliorates skin inflammation, targeting key cytokines and signaling pathways. Additionally, our results revealed that topically administered RGRN-305 was effective, suggesting it may be a topical medication (new route of administration). Our basic scientific findings of HSP90 inhibition may be translated to a potential novel treatment for inflammatory skin diseases, providing a scientific basis for further clinical evaluation that hopefully may benefit patients.

## Data availability statement

The data is publicily available at Gene Expression Omnibus (https://www.ncbi.nlm.nih.gov/geo/query/acc.cgi?acc=GSE222000; accession number GSE222000).

## Ethics statement

The studies involving human participants were reviewed and approved by The Central Jutland Regional Committee on Health Research Ethics. The patients/participants provided their written informed consent to participate in this study. The animal study was reviewed and approved by The Danish Animal Experiments Inspectorate.

## Author contributions

Conceptualization: HB, AB, GG, LI, and CJ. Formal analysis: HB and SS; funding acquisition: HB, LI, and CJ. Investigation: HB and SS. Methodology: HB, AB, GG, LK, LI, and CJ. Project administration: HB, AB, LI, and CJ. Resources: HB, LK, LI, and CJ. Supervision: LK, CJ, and LI. Visualization: HB. Writing - original draft preparation: HB. Writing - review and editing: HB, SS, AB, GG, LK, LI, and CJ. All authors contributed to the article and approved the submitted version.

## References

[1] Medzhitov R. Origin and physiological roles of inflammation. Nature (2008) 454(7203):428–35. doi: 10.1038/nature07201 18650913

[2] Furman D Campisi J Verdin E Carrera-Bastos P Targ S Franceschi C. Chronic inflammation in the etiology of disease across the life span. Nat Med (2019) 25(12):1822–32. doi: 10.1038/s41591-019-0675-0 PMC7147972 31806905

[3] Wolk K Join-Lambert O Sabat R. Aetiology and pathogenesis of hidradenitis suppurativa. Br J Dermatol (2020) 183(6):999–1010. doi: 10.1111/bjd.19556 33048349

[4] Armstrong AW Puig L Joshi A Skup M Williams D Li J. Comparison of biologics and oral treatments for plaque psoriasis: A meta-analysis. JAMA Dermatol (2020) 156(3):258–69. doi: 10.1001/jamadermatol.2019.4029 PMC7042876 32022825

[5] Ben Abdallah H Emmanuel T Bregnhøj A Johansen C Iversen L. Early intervention and disease memory in psoriasis: A literature review. JEADV Clin Pract (2022) 1(4):307–316. doi: 10.1002/jvc2.63

[6] Ben Abdallah H Johansen C Iversen L. Key signaling pathways in psoriasis: Recent insights from antipsoriatic therapeutics. Psoriasis (Auckl) (2021) 11:83–97. doi: 10.2147/ptt.S294173 34235053 PMC8254604

[7] Hartl FU Bracher A Hayer-Hartl M. Molecular chaperones in protein folding and proteostasis. Nature (2011) 475(7356):324–32. doi: 10.1038/nature10317 21776078

[8] Sager RA Khan F Toneatto L Votra SD Backe SJ Woodford MR. Targeting extracellular Hsp90: A unique frontier against cancer. Front Mol Biosci (2022) 9:982593. doi: 10.3389/fmolb.2022.982593 36060252 PMC9428293

[9] Schopf FH Biebl MM Buchner J. The Hsp90 chaperone machinery. Nat Rev Mol Cell Biol (2017) 18(6):345–60. doi: 10.1038/nrm.2017.20 28429788

[10] Zuehlke A Johnson JL. Hsp90 and Co-chaperones twist the functions of diverse client proteins. Biopolymers (2010) 93(3):211–7. doi: 10.1002/bip.21292 PMC2810645 19697319

[11] Lang BJ Guerrero ME Prince TL Okusha Y Bonorino C Calderwood SK. The functions and regulation of heat shock proteins; key orchestrators of proteostasis and the heat shock response. Arch Toxicol (2021) 95(6):1943–70. doi: 10.1007/s00204-021-03070-8 34003342

[12] Prodromou C. Mechanisms of Hsp90 regulation. Biochem J (2016) 473(16):2439–52. doi: 10.1042/bcj20160005 PMC4980810 27515256

[13] Taipale M Krykbaeva I Koeva M Kayatekin C Westover KD Karras GI. Quantitative analysis of Hsp90-client interactions reveals principles of substrate recognition. Cell (2012) 150(5):987–1001. doi: 10.1016/j.cell.2012.06.047 22939624 PMC3894786

[14] Tukaj S Węgrzyn G. Anti-Hsp90 therapy in autoimmune and inflammatory diseases: A review of preclinical studies. Cell Stress Chaperones (2016) 21(2):213–8. doi: 10.1007/s12192-016-0670-z PMC4786535 26786410

[15] Picard D. Hsp90 interactors (2022). Available at: https://www.picard.ch/ https://www.picard.ch/downloads/Hsp90interactors.pdf.

[16] Rice JW Veal JM Fadden RP Barabasz AF Partridge JM Barta TE. Small molecule inhibitors of Hsp90 potently affect inflammatory disease pathways and exhibit activity in models of rheumatoid arthritis. Arthritis Rheum (2008) 58(12):3765–75. doi: 10.1002/art.24047 19035474

[17] Yun TJ Harning EK Giza K Rabah D Li P Arndt JW. Ec144, a synthetic inhibitor of heat shock protein 90, blocks innate and adaptive immune responses in models of inflammation and autoimmunity. J Immunol (2011) 186(1):563–75. doi: 10.4049/jimmunol.1000222 21131419

[18] Shimp SK 3rd Chafin CB Regna NL Hammond SE Read MA Caudell DL. Heat shock protein 90 inhibition by 17-dmag lessens disease in the Mrl/Lpr mouse model of systemic lupus erythematosus. Cell Mol Immunol (2012) 9(3):255–66. doi: 10.1038/cmi.2012.5 PMC4012849 22543833

[19] Tukaj S Tiburzy B Manz R de Castro Marques A Orosz A Ludwig RJ. Immunomodulatory effects of heat shock protein 90 inhibition on humoral immune responses. Exp Dermatol (2014) 23(8):585–90. doi: 10.1111/exd.12476 24961936

[20] Kasperkiewicz M Müller R Manz R Magens M Hammers CM Somlai C. Heat-shock protein 90 inhibition in autoimmunity to type vii collagen: Evidence that nonmalignant plasma cells are not therapeutic targets. Blood (2011) 117(23):6135–42. doi: 10.1182/blood-2010-10-314609 21490339

[21] Stenderup K Rosada C Gavillet B Vuagniaux G Dam TN. Debio 0932, a new oral Hsp90 inhibitor, alleviates psoriasis in a xenograft transplantation model. Acta Derm Venereol (2014) 94(6):672–6. doi: 10.2340/00015555-1838 24604074

[22] Bhatia A O'Brien K Guo J Lincoln V Kajiwara C Chen M. Extracellular and non-chaperone function of heat shock protein-90α is required for skin wound healing. J Invest Dermatol (2018) 138(2):423–33. doi: 10.1016/j.jid.2017.08.043 PMC6559362 28942361

[23] Cheng CF Sahu D Tsen F Zhao Z Fan J Kim R. A fragment of secreted Hsp90α carries properties that enable it to accelerate effectively both acute and diabetic wound healing in mice. J Clin Invest (2011) 121(11):4348–61. doi: 10.1172/jci46475 PMC3204835 22019588

[24] Bao R Lai CJ Qu H Wang D Yin L Zifcak B. Cudc-305, a novel synthetic Hsp90 inhibitor with unique pharmacologic properties for cancer therapy. Clin Cancer Res (2009) 15(12):4046–57. doi: 10.1158/1078-0432.Ccr-09-0152 19509149

[25] Hansen RS Thuesen KKH Bregnhøj A Moldovan LI Kristensen LS Grek CL. The Hsp90 inhibitor rgrn-305 exhibits strong immunomodulatory effects in human keratinocytes. Exp Dermatol (2021) 30(6):773–781. doi: 10.1111/exd.14302 33583094

[26] Bregnhøj A Thuesen KKH Emmanuel T Litman T Grek CL Ghatnekar GS. Hsp90 inhibitor rgrn-305 for oral treatment of plaque-type psoriasis: Efficacy, safety and biomarker results in an open-label proof-of-Concept study. Br J Dermatol (2021) 186(5):861–874. doi: 10.1111/bjd.20880 34748646

[27] Wu BC Skovbakke SL Masoudi H Hancock REW Franzyk H. *In vivo* anti-inflammatory activity of lipidated peptidomimetics Pam-(Lys-Bnspe)(6)-Nh(2) and lau-(Lys-Bnspe)(6)-Nh(2) against pma-induced acute inflammation. Front Immunol (2020) 11:2102. doi: 10.3389/fimmu.2020.02102 32983167 PMC7485003

[28] Zhang G Liu X Wang C Qu L Deng J Wang H. Resolution of pma-induced skin inflammation involves interaction of ifn-Γ and Alox15. Mediators Inflammation (2013) 2013:930124. doi: 10.1155/2013/930124 PMC3683498 23818745

[29] Johansen C. Generation and culturing of primary human keratinocytes from adult skin. J Vis Exp (2017) 130. doi: 10.3791/56863 PMC5755678 29286419

[30] Biosystems A. Applied biosystems stepone™ and steponeplus™ real-time pcr systems: Applied biosystems (2010). Available at: https://tools.thermofisher.com/content/sfs/manuals/cms_046739.pdf.

[31] Benjamini Y Krieger AM Yekutieli D. Adaptive linear step-up procedures that control the false discovery rate. Biometrika (2006) 93(3):491–507. doi: 10.1093/biomet/93.3.491

[32] Liao Y Smyth GK Shi W. The r package rsubread is easier, faster, cheaper and better for alignment and quantification of rna sequencing reads. Nucleic Acids Res (2019) 47(8):e47. doi: 10.1093/nar/gkz114 30783653 PMC6486549

[33] Love MI Huber W Anders S. Moderated estimation of fold change and dispersion for rna-seq data with Deseq2. Genome Biol (2014) 15(12):550. doi: 10.1186/s13059-014-0550-8 25516281 PMC4302049

[34] Ge SX Jung D Yao R. Shinygo: A graphical gene-set enrichment tool for animals and plants. Bioinformatics (2020) 36(8):2628–9. doi: 10.1093/bioinformatics/btz931 PMC7178415 31882993

[35] Li L Chen NN You QD Xu XL. An updated patent review of anticancer Hsp90 inhibitors (2013-present). Expert Opin Ther Pat (2021) 31(1):67–80. doi: 10.1080/13543776.2021.1829595 32990109

[36] Wang X Ju W Renouard J Aden J Belinsky SA Lin Y. 17-Allylamino-17-Demethoxygeldanamycin synergistically potentiates tumor necrosis factor-induced lung cancer cell death by blocking the nuclear factor-kappab pathway. Cancer Res (2006) 66(2):1089–95. doi: 10.1158/0008-5472.Can-05-2698 16424045

[37] Broemer M Krappmann D Scheidereit C. Requirement of Hsp90 activity for ikappab kinase (Ikk) biosynthesis and for constitutive and inducible ikk and nf-kappab activation. Oncogene (2004) 23(31):5378–86. doi: 10.1038/sj.onc.1207705 15077173

[38] Ghoreschi K Balato A Enerbäck C Sabat R. Therapeutics targeting the il-23 and il-17 pathway in psoriasis. Lancet (London England) (2021) 397(10275):754–66. doi: 10.1016/s0140-6736(21)00184-7 33515492

[39] Calderwood SK Gong J Murshid A. Extracellular hsps: The complicated roles of extracellular hsps in immunity. Front Immunol (2016) 7:159. doi: 10.3389/fimmu.2016.00159 27199984 PMC4842758

[40] Poggio P Sorge M Seclì L Brancaccio M. Extracellular Hsp90 machineries build tumor microenvironment and boost cancer progression. Front Cell Dev Biol (2021) 9:735529. doi: 10.3389/fcell.2021.735529 34722515 PMC8551675

[41] Tukaj S Kleszczyński K Vafia K Groth S Meyersburg D Trzonkowski P. Aberrant expression and secretion of heat shock protein 90 in patients with bullous pemphigoid. PloS One (2013) 8(7):e70496. doi: 10.1371/journal.pone.0070496 23936217 PMC3728143

