# Supplementary material for: Heat shock protein 90 inhibitor RGRN-305 potently attenuates skin inflammation
Source: Front Immunol. 2023 Feb 7;14:1128897. doi: 10.3389/fimmu.2023.1128897 (PMC9941631; doi:10.3389/fimmu.2023.1128897)
Supplement: Supplementary file 1 [file DataSheet_1.docx]

**Figure S1**


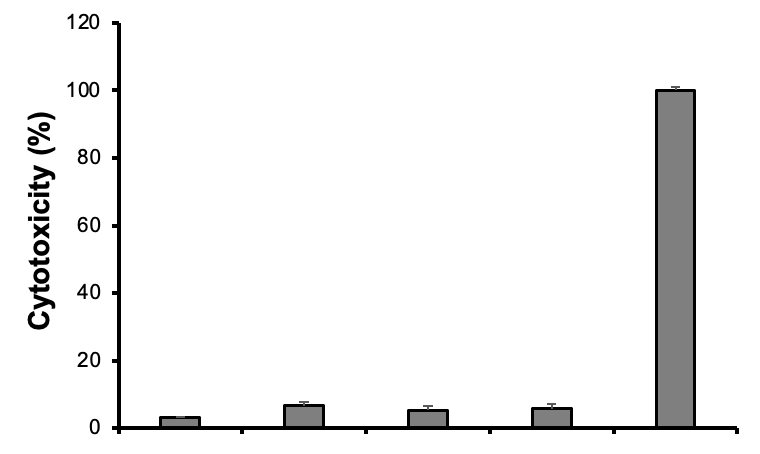


Vehicle

TPA

RGRN-305

RGRN-305+TPA

Pos. con.

**Figure S1 Lactate dehydrogenase (LDH) cytotoxicity assay in primary human keratinocytes.** The keratinocytes were cultured in a 96-well plate and preincubated with 10 µM RGRN-305 for 8 hours before stimulation with TPA (100 nM) or DMSO for 24 hours. The activity of LDH was measured following the protocol by the manufacturer (LDH-Glo^TM^ cytotoxicity assay; Promega, Madison, WI, USA). The absorbance was measured at 490 nm using a microplate reader (Fluoroskan Ascent FL). Triton X-100 was used as a positive control (pos. con.) and was set to 100% cell death.

Abbreviations: LDH, Lactate dehydrogenase. TPA, 12-O-Tetradecanoylphorbol-13-acetate.
