# Supplementary material for: Heat shock protein 90 inhibitor RGRN-305 potently attenuates skin inflammation
Source: Front Immunol. 2023 Feb 7;14:1128897. doi: 10.3389/fimmu.2023.1128897 (PMC9941631; doi:10.3389/fimmu.2023.1128897)
Supplement: Supplementary file 2 [file DataSheet_2.docx]

**
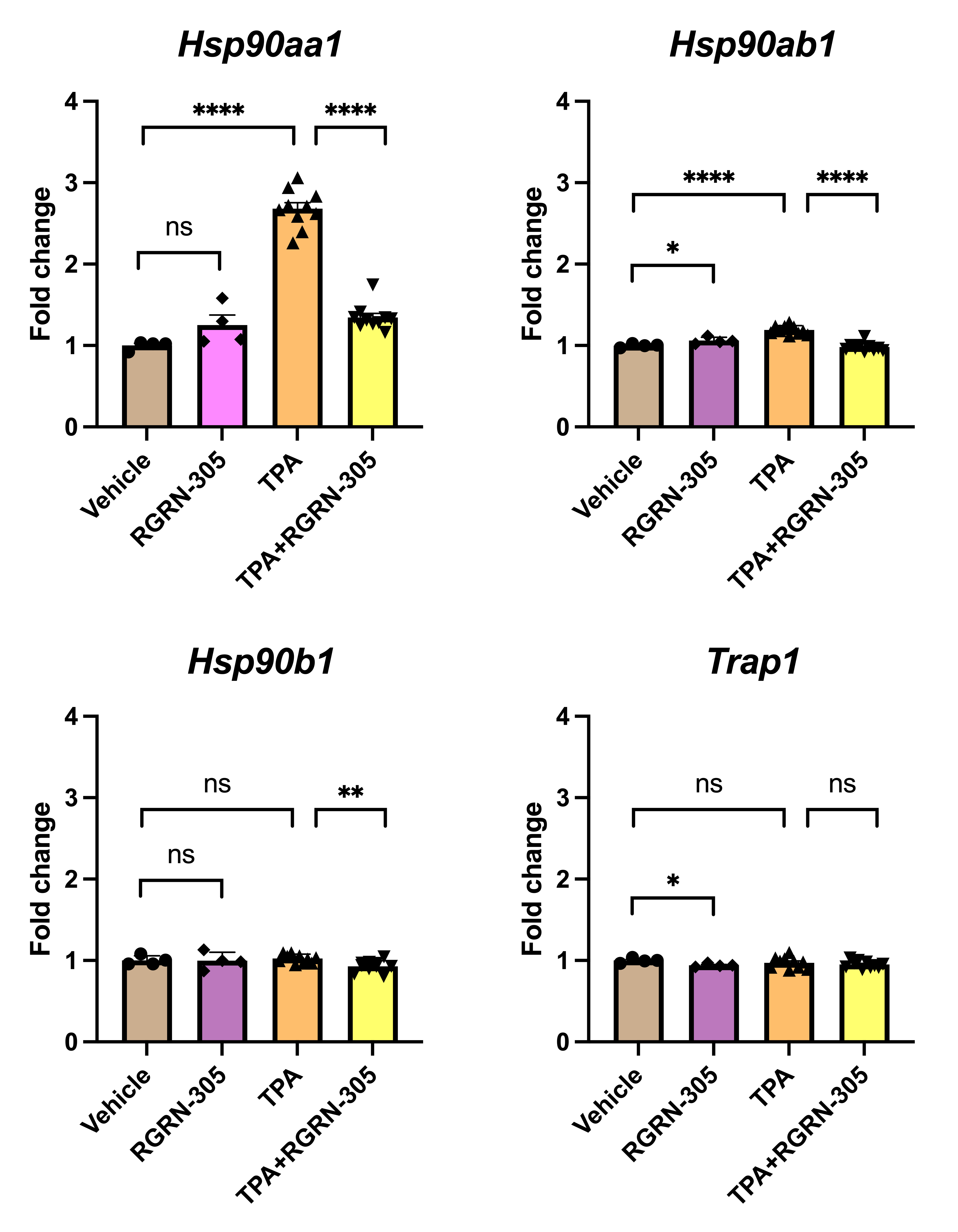
Figure S2 Gene expression of Hsp90 genes in mice challenged with TPA and pretreated with RGRN-305.** Number of mice in treatment groups: vehicle = 4, RGRN-305 = 4, TPA = 10, TPA+RGRN-305 = 10.

Gene expression levels were determined by RNA sequencing. Data are shown as mean ± SEM fold change relative to the vehicle. Unpaired t-tests were used for pairwise comparisons.* p < 0.05, ** p ≤0.01, *** p ≤0.001, **** p ≤ 0.0001.

Abbreviations: TPA, 12-O-Tetradecanoylphorbol-13-acetate.
