# Supplementary material for: Heat shock protein 90 inhibitor RGRN-305 potently attenuates skin inflammation
Source: Front Immunol. 2023 Feb 7;14:1128897. doi: 10.3389/fimmu.2023.1128897 (PMC9941631; doi:10.3389/fimmu.2023.1128897)
Supplement: Supplementary file 4 [file DataSheet_4.docx]

**
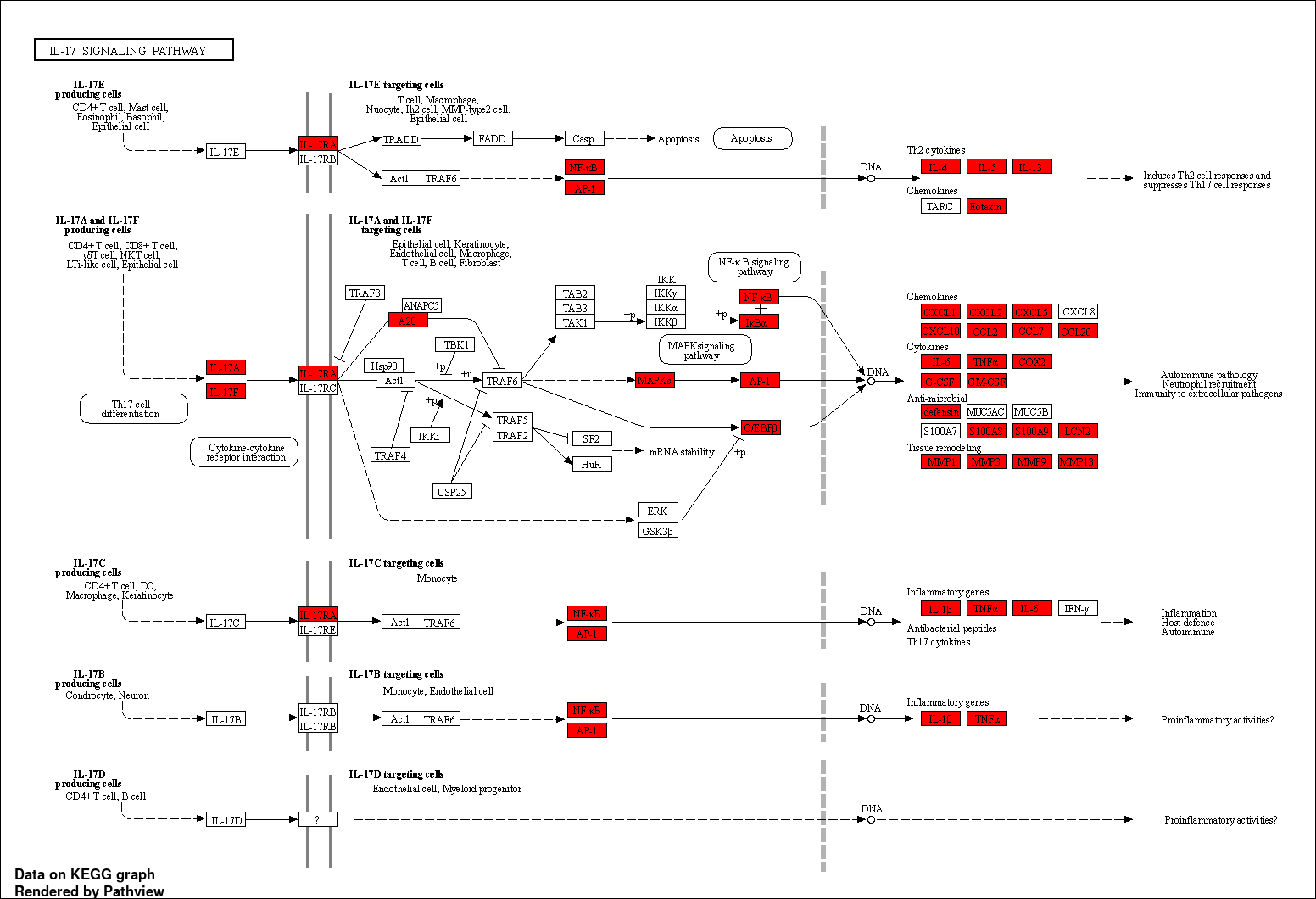
**

**Figure S4 Effects of RGRN-305 on ‘IL-17 signaling’ KEGG pathway.** The pathway diagram illustrates downregulated differential expressed genes colored in red.
